# Phosphoproteome Analysis Using Two-Dimensional Electrophoresis Coupled with Chemical Dephosphorylation

**DOI:** 10.3390/foods11193119

**Published:** 2022-10-07

**Authors:** Raquel Rodríguez-Vázquez, Daniel Mouzo, Carlos Zapata

**Affiliations:** Department of Zoology, Genetics and Physical Anthropology, University of Santiago de Compostela, 15872 Santiago de Compostela, Spain

**Keywords:** hydrogen fluoride-pyridine, meat phosphoproteome, phosphorylation, phosphorylation rate, post-translational protein modification

## Abstract

Protein phosphorylation is a reversible post-translational modification (PTM) with major regulatory roles in many cellular processes. However, the analysis of phosphoproteins remains the most challenging barrier in the prevailing proteome research. Recent technological advances in two-dimensional electrophoresis (2-DE) coupled to mass spectrometry (MS) have enabled the identification, characterization, and quantification of protein phosphorylation on a global scale. Most research on phosphoproteins with 2-DE has been conducted using phosphostains. Nevertheless, low-abundant and low-phosphorylated phosphoproteins are not necessarily detected using phosphostains and/or MS. In this study, we report a comparative analysis of 2-DE phosphoproteome profiles using Pro-Q Diamond phosphoprotein stain (Pro-Q DPS) and chemical dephosphorylation of proteins with HF-P from *longissimus thoracis* (LT) muscle samples of the Rubia Gallega cattle breed. We found statistically significant differences in the number of identified phosphoproteins between methods. More specifically, we found a three-fold increase in phosphoprotein detection with the HF-P method. Unlike Pro-Q DPS, phosphoprotein spots with low volume and phosphorylation rate were identified by HF-P technique. This is the first approach to assess meat phosphoproteome maps using HF-P at a global scale. The results open a new window for 2-DE gel-based phosphoproteome analysis.

## 1. Introduction

Protein post-translational modifications (PTMs) play a key functional role in the proteome complexity including activity, interaction and localization of proteins [1]. Phosphorylation is the most important PTM which modulates many cellular processes, metabolism, biosignaling networks and molecular interactions [2]. In particular, the study of phosphoproteome helps to unravel the biochemical processes underlying food quality, as in the case of meat, where phosphoproteomic changes have been reported to lead to variations in meat tenderness [3]. Although advances in proteomic methodologies have made it possible to increase the effectiveness of phosphoproteome studies, several issues remain a challenge for a comprehensive study of phosphorylation [4].

In the last years, several strategies have been developed to quantify the degree of protein phosphorylation. The most commonly used methods are Western blot [5], radioisotopes [6]; two-dimensional electrophoresis (2-DE) together with phosphospecific stains [7] and gel-free coupled with mass spectrometry (MS) [7]. More specifically, in recent years, there have been great developments in gel-free methodologies facilitating proteome study on a large-scale quantitative, site-specific and sensitive measurement of protein phosphorylation [4]. However, MS faces important challenges in the phosphoproteomic field, including low-abundant phosphoproteins, low-phosphorylation stoichiometry, high dynamic range [4,7], non-validation of phosphorylation sites in public databases that contributes to the accumulation of false positives [4] and difficulty in identifying and quantifying differentially phosphorylated protein isoforms [8]. For all these reasons, although gel-free methods for protein phosphorylation are trying to outpace 2-DE, this technique still plays an important role in this field and can settle some difficulties that are present in gel-free methods. One of the advantages of 2-DE technology is its ability to identify small changes in the abundance of proteoforms generated by phosphorylations and a very precise separation of entire proteins allowing quantitative evaluation of proteoforms that could become innovated in gel-free methods as a result of protein digestion [9,10,11].

The 2-DE coupled with MS approach is one of the most used and reproducible proteomics techniques [12]. This method allows the analysis of complex protein mixtures, providing a snapshot of the proteome in the sample of study [13]. The 2-DE has traditionally been used to separate proteins regarding their isoelectric point (p*I*) and relative molecular mass (*M*r) [14]. New strengths of 2-DE have been developed over the past few years such as a new measure of quantitative proteomic distance [15] and other applications reviewed by Lee et al. [11] and Oliveira et al. [9]. The p*I* of phosphorylated proteins is altered by isoforms with different charges (negative or positive) in amino acids or with the addition of negatively charged phosphate groups replacing hydroxyl groups in amino acid residues [16]. Unlike gel-free methods, differentially charged isoforms can be successfully identified as chains of horizontally lengthened spots in the first dimension (isoelectric focusing) on 2-DE gels [17,18]. In recent years, fluorescent stains have been applied for the detection of protein phosphorylation. One such stain is Pro-Q DPS [19], which allows in-gel identification of phosphoserine, phosphothreonine and phosphotyrosine residues [20]. Its detection limit was established as a few nanograms and has a linear signal intensity [20,21]. Nevertheless, this stain presents some drawbacks, including low sensitivity, which implies that the low-abundant phosphoproteins cannot be detected [21]; high cost of the reagent [22]; the appearance of false positives [23] and the minimal number of phosphorylated sites per protein to detect PTMs is unknown [24].

Protein dephosphorylation is an alternative strategy for deciphering the phosphoproteome. Numerous studies have used enzymatic dephosphorylation with well-characterized enzymes that are capable of removing phosphate groups from proteins [25,26,27,28,29]. However, there are some drawbacks such as this type of dephosphorylation is dependent on phosphatase preferences, i.e., sequence similarity of the catalytic domain [30]; enzyme cleavage sites located too close to phosphorylated residues could decrease cleavage efficiency [31] or inhibition of phosphatase activity during sample processing, leading to partial dephosphorylation [32,33]. This limitation can be circumvented by applying chemical dephosphorylation with hydrogen fluoride-pyridine (HF-P). Kuyama et al. [32] developed an efficient method for the removal of phosphate moieties from proteins with HF-P. Kita et al. [34] adapted this method for the study of phosphorylation of glucose-regulated protein 58 in rat liver using 2-DE coupled to MS. More recently, the method of chemical dephosphorylation with HF-P was successfully used in targeted 2-DE-based proteomic research for complete characterization of multiple phosphorylated isoforms of plant storage proteins (phaseolin and patatin) [35,36,37]. To our knowledge, HF-P has not yet been applied in multiplex phosphoprotein analyses. In this work, we use for the first time the method of chemical dephosphorylation with HF-P coupled to high-resolution 2-DE multiplex phosphoprotein analysis. This approach could have potential application in the industry and, more specifically, in meat science to obtain phospho-biomarkers to investigate food authenticity, meat quality, food toxicity or adulteration in meat, among others [38,39,40]. This new approach applied to meat science or food science could be a key tool to increase knowledge about food products.

The aim of the present study was to contrast the global phosphoproteome of meat using different approaches through 2-DE gels stained with the phosphostain Pro-Q DPS [3,41] and through chemical dephosphorylation of proteins employing HF-P [35]. The phosphorylation rate (*PR*) of phosphoproteins in the *longissimus thoracis* (LT) muscle of the Rubia Gallega bovine breed (*Bos taurus*), which is one of the major cattle autochthon breeds in the Spanish meat industry [42], was evaluated using both methods. In particular, the results of this research suggest that the HF-P method of dephosphorylation would be a useful tool for detecting and quantifying the phosphorylation state of proteins.

## 2. Materials and Methods

**Figure 1:**
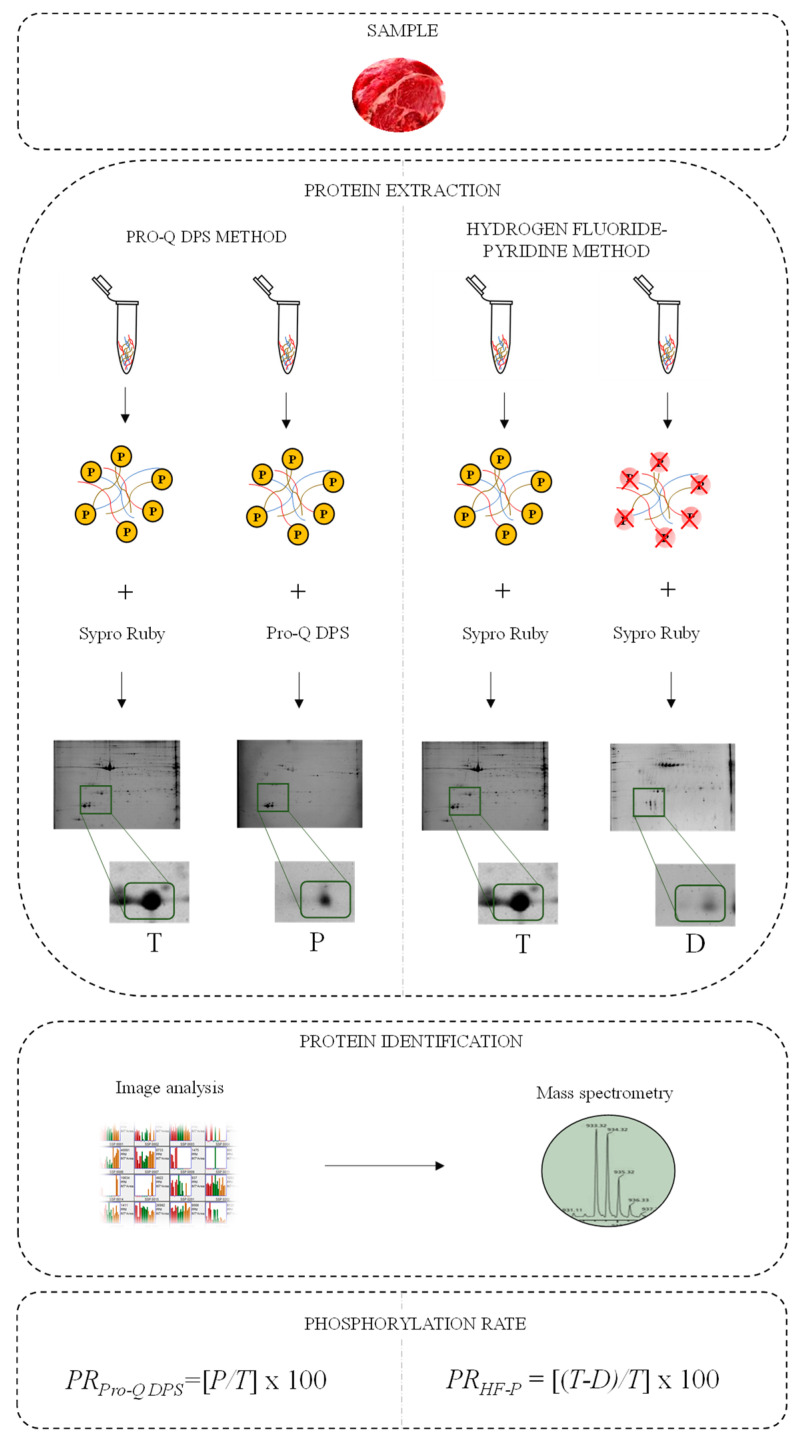
Workflow and experimental design of the quantitative phosphoproteomic analysis of meat proteins on 2-DE gels from LT muscle by Pro-Q DPS and HF-P methods. Meat proteins were separated by 2-DE and visualized with Sypro Ruby and the gel image was subsequently scanned to obtain meat protein reference patterns and total protein volume of spots (*T*). Phosphorylated patterns were assessed from untreated sample stained with Pro-Q DPS stain that binds to phosphate groups to identify phosphorylated proteins and consequently obtain the phosphorylated spot volumes (*P*). Treatment of chemical dephosphorylation with HF-P following staining with Sypro Ruby was performed to obtain the pattern of dephosphorylated proteins and their volumes (*D*). Individual protein spots of gels were selected, extracted from gels and identified by MALDI-TOF and MALDI-TOF/TOF MS. Pro-Q DPS and HF-P methods were used to evaluate the phosphorylation rate (*PR*).

An overview of the phosphoproteomic strategies and experimental procedures to evaluate the phosphorylation rate of meat proteins is described in Figure 1.

### 2.1. Animal Material

Proteomic analyses were obtained from meats of male calves of the Rubia Gallega bovine breed (*Bos taurus*) (Spain). Briefly, male calves aged 5–6 months were transported from family farms to an accredited abattoir (Lugo, Spain), stunned, slaughtered and dressed following the current European Union regulations (Council Directive 93/119/EEC). Meat samples (steak of 2 cm from each animal) were excised 2 h post mortem from LT muscle at the 13th rib position, lyophilized and frozen at −80 °C until the time of protein extraction. Three independent biological replicates of Rubia Gallega were used for the proteomic analyses.

### 2.2. Protein Extraction and Quantification

Total proteins were extracted from 50 mg of lyophilized tissue. Protein extracts were transferred to 1.5 mL tubes containing lysis buffer (4% CHAPS; 2 M thiourea; 7 M urea; 10 mM dithiothreitol (DTT); 2% Pharmalyte pH 3–10) (GE Healthcare, Uppsala, Sweden) for 2 h at 25 °C. Sonifier 250 (Branson Ultrasonics, Danbury, CT, USA) was used to lyse an aliquot of 250 µL. Protein purification and extraction were performed with Clean-Up Kit (GE Healthcare) removing any interfering substances as stated in manufacturer’s instructions (GE Healthcare) and then resuspended in 500 µL of lysis buffer. Protein concentration was assessed for each sample using the CB-X protein assay kit (G-Biosciences, St. Louis, MO, USA) detailed in manufacturer’s recommendations and using a microplate reader, Chromate 4300 (Awareness Technology, Palm City, FL, USA).

### 2.3. Two-Dimensional Electrophoresis (2-DE)

High-resolution 2-DE was performed according to Görg et al. [43] with some modifications as previously described by Franco et al. [41]. The first dimension was run in 24 cm long pH 4–7 linear gradient ReadyStrip^TM^ IPG Strips (Bio-Rad Laboratories, Hercules, CA, USA), loaded with 450 µg of protein from each sample, concurrently with 0.6% DTT and 1% immobilized pH gradient (IPG) buffer (Bio-Rad Laboratories). Isoelectric focusing (IEF) was performed on PROTEAN IEF Cell (Bio-Rad Laboratories). Initially, 50 V were applied for 12 h to rehydrate each strip, following a voltage grade until 70 kWh. Subsequently, strips were immersed in equilibration buffer I (50 mM Tris pH 8.8, 30% glycerol, 2% SDS, 6 M urea and 1% DTT) for 15 min at room temperature and in the same conditions with equilibration buffer II (50 mM Tris pH 8.8, 30% glycerol, 2% SDS, 6 M urea and 2.5% iodoacetamide). For the second dimension, proteins were resolved on 13% SDS-PAGE gels of 24 × 20 cm using Ettan DALTsix vertical system (GE Healthcare).

### 2.4. Detection of Phosphoproteins and Total Proteins

Gels were stained with phosphoprotein-specific fluorescent dye Pro-Q DPS (Thermo Fisher Scientific, Waltham, MA, USA) according to Agrawal and Thelen, with some modifications [21,44]. The 2-DE gels were covered twice with a fixation solution (50% methanol and 10% acetic acid, for 30 min) and washed twice with distilled water for 15 min per wash. The gels were then incubated two-fold with water-diluted Pro-Q DPS (120 min). For removal of gel-bound nonspecific Pro-Q DPS, gels were distained four times with distaining solution (20% acetonitrile pH 4.0 and 50 mM sodium acetate) for 30 min, and washed with distilled water (twice, 5 min per wash). The PeppermintStick^TM^ (Thermo Fisher Scientific) phosphoprotein marker was added to meat protein extract previously to 2-DE. The molecular mass standards of PeppermintStick^TM^ contain two phosphorylated (ovalbumin of 45.0 kDa and β-casein of 23.6 kDa) and four unphosphorylated (β-galactosidase of 116.25 kDa, bovine serum albumin of 66.2 kDa, avidin of 18.0 kDa and lysozyme of 14.4 kDa) proteins. The same gels were post-stained with Sypro Ruby (Lonza, Rockland, ME, USA) stain as described in manufacturer’s indications.

### 2.5. Image Analysis

Images of 2-DE gels were acquired with Gel Doc^TM^ XR + Imaging System (Bio-Rad Laboratories) and digitalized gels were analyzed through PDQuest Advanced software version 8.0.1 (Bio-Rad Laboratories, Hercules, CA, USA) [45]. Spot volumes were identified, matched across biological replicates and quantified following background noise subtraction and normalization from total density of valid spots. For image analysis, spots detected in at the least two of three biological replicates were included. Experimental isoelectric point (p*I*) values of protein spots were assessed from their 2-DE gel position relative to linear gradient pH 4–7 focused strips, while experimental *M_r_* values were obtained with molecular mass markers from 15 to 200 kDa (Fermentas, Ontario, ON, Canada).

### 2.6. Protein Dephosphorylation

Chemical dephosphorylation of protein extracts was performed using HF-P as previously described by Kuyama et al. [32], with some modifications [35]. For each meat sample, 1 mg of total protein extract was dissolved in 250 µL of HF-P and placed in an ice bath for 2 h. Following this, 10 M NaOH was used to neutralize the solution, desalinated by using Amicon Ultra-4 centrifugal filter devices (Millipore, MA, USA) and then eluted in 300 µL of lysis buffer. Subsequently, Clean-up kit (GE Healthcare) was used twice for protein purification. The evaluation of dephosphorylation efficiency was performed using the ovalbumin phosphoprotein marker (45.0 kDa, Molecular Probes, Leiden, The Netherlands) was used. Protein quantification of total protein was assessed by the commercial CB-X protein assay kit (G-Bioscience). The changes obtained by dephosphorylations were identified by 2-DE as mentioned above, with SYPRO stain. Gel images were scanned with Gel Doc XR+ Imaging System (Bio-Rad Laboratories) and analyzed by PDQuest software.

### 2.7. Mass Spectrometry (MS) Analysis

Protein identification was performed by MALDI-TOF and MALDI-TOF/TOF MS as described by Franco et al. [41]. Selected spots were excised from gels and were subsequently in-gel digested with modified trypsin (Promega, Madison, WI, USA). The resulting peptides were dried using a SpeedVac (Thermo Fisher Scientific) and stored at −20 °C. After that, 4 µL 0.5% formic acid was used to resolubilize the dried peptide samples and then mixed with the MALDI matrix solution (0.5 µL), containing 3 mg α-Cyano-4- hydroxycinnamic acid (CHCA) dissolved in 1 mL of acetonitrile [ACN] (50%) and trifluoroacetic acid [TFA] (0.1%). The mixture was placed onto a 384 Opti-TOF MALDI plate (Applied Biosystems, Foster City, CA, USA) by the thin layer method. MS spectra were obtained in positive-ion reflector mode with a Nd:YAG, 355 nm wavelength laser, an average of 1000 laser shots and at least three trypsin autolysis peaks were used for internal calibration. Mass spectrometric data were obtained in a 4800 MALDI-TOF/TOF mass spectrometer (Applied Biosystems). All MSMS spectra were performed by selecting the precursors with a relative resolution of 300 (FWHM) and metastable suppression. Mass spectra of samples were achieved using 4000 Series Explorer Software v. 3.5 (Applied Biosystems). Peptide mass-fingerprinting (PMF) and MSMS fragmentation spectra data of each sample were combined through the GPS Explorer Software version 3.6 using Mascot software v. 2.1 (Matrix Science, Boston, MA, USA) to search against the B. taurus UniProtKB/Swiss-Prot databases (accessed on 1 September 2019). Parameters of Mascot software were: 30 ppm of precursor tolerance, allowance one missed cleavage site, 0.35 Da of fragment mass tolerance, carbamidomethyl cysteine and oxidized methionine as fixed and variable modification, respectively. All spectra and identifications were manually checked. Protein scores higher than 97 were used for assessing statistical significance (*p*-value < 0.05).

### 2.8. Statistical Analysis

The *PR* for each protein spot on 2-DE gels stained with Pro-Q DPS was calculated by the ratio *PR_Pro-Q DPS_ =* [*P/T*] × 100, *P* and *T* being the volumes of the same spot on gels stained with Pro-Q DPS and Sypro Ruby, respectively [3]. The *p*-values > *T*-values were represented with a *PR_Pro-Q DPS_ =* 100. On the other hand, the phosphorylation rate of protein spots treated with HF-P was estimated by the coefficient *PR_HF-P_* = [(*T − D)/T*] × 100, in which *T* and *D* are the volumes of spots untreated (total protein volume) and treated (dephosphorylated protein volumes) with HF-P, respectively [35]. Non-parametric bootstrap (95%) confidence intervals (CIs) were computed for mean values of *PR_Pro-Q DPS_* and *PR_HF-P_* across three biological replicates [41]. For each mean value, 2000 bootstrap samples of size *N =* 3 were drawn applying a Monte Carlo algorithm. Bootstrap CIs were corrected by the Bonferroni correction for multiple comparisons, after bias correction by percentile method using the theoretical normal distribution [46]. Statistically significant differences in the total number of phosphorylated protein spots identified with both methods were assessed with the Fisher’s exact test using XLSTAT software version 2014.5.03 (Addinsoft, Andernach, Germany).

## 3. Results and Discussion

### 3.1. Phosphoproteome Map: An Overview

**Figure 2:**
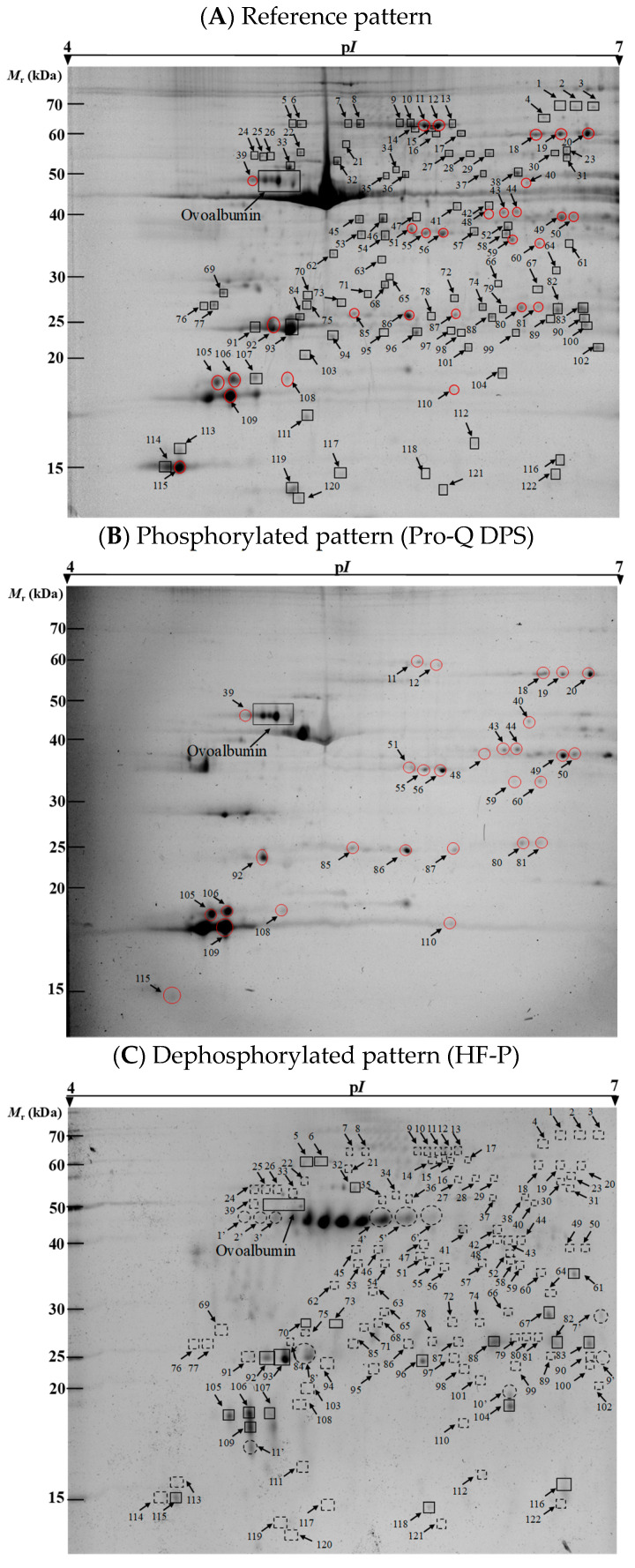
Representative 2-DE gels of meat proteins from LT muscle obtained for three different protocols. (**A**) The 2-DE reference pattern of total protein obtained from untreated samples. Gels were stained with Sypro Ruby stain. (**B**) The 2-DE phosphorylated pattern after staining with the phosphoprotein-specific fluorescent dye Pro-Q DPS. (**C**) The 2-DE dephosphorylated pattern after HF-P treatment and staining with Sypro Ruby. Protein spots with statistically significant differences in phosphorylation rates between both methods (Pro-Q DPS and HF-P) are marked and numbered. Red circles represent phosphorylated protein spots detected by Pro-Q DPS method and with statistically significant differences in *PR* between both methods. Dashed squares are missing and circles are newly arisen spots after dephosphorylation, with regard to reference profiles.

Representative reference (Sypro Ruby stain), phosphorylated (Pro-Q DPS) and dephosphorylated (HF-P) 2-DE proteomic profiles of meat samples at 2 h post mortem from LT bovine muscle of the Rubia Gallega breed are shown in Figure 2. Identification, matching and assessment of spot volumes for each biological replicate on 2-DE gels were performed using PDQuest software. A total of 174 individual protein spots were matched among reference, phosphorylated and dephosphorylated profiles on 2-DE gels. 

Reference and phosphoproteome profiles of meat samples obtained by Sypro Ruby Stain and Pro-Q DPS, respectively, are shown in Figure 2A,B. Pro-Q DPS is a phosphoprotein specific dye which detects stable phosphorylated proteins on 2-DE gels [47]. PeppermintStick phosphoprotein marker (containing the phosphoprotein ovalbumin) validated the specificity of the identification of phosphoproteins by Pro-Q DPS, as reflected in other studies [3,37]. We also decided to investigate alternative methods to visualize phosphoproteins through 2-DE (Figure 2C) because Pro-Q DPS identified a scarce number of phosphorylated proteins. HF-P is a novel method suitable for monitoring the phosphorylation status of proteins directly in polyacrylamide gels and allows sensitive detection of phosphoproteins in two-dimensional gels [35,37]. This approach is one of the best chemical dephosphorylated-based detection systems for the specific and sensitive analysis of protein- and peptide-phosphorylation status [32]. Note that the ovalbumin phosphoprotein marker was also used in the method of chemical dephosphorylation with HF-P to assess the efficiency of this method (Figure 2C). As a result of the loss of phosphate groups, the ovalbumin spots shifted to more basic gel positions or disappeared after HF-P treatment, in accordance with Bernal et al. [37]. It is remarkable that the percentage of phosphorylated protein spots identified on 2-DE gels with HF-P method was 87.4% (152 out of 174 total spots), while only 25.3% (44 out of 174 spots) was identified with Pro-Q DPS. These results show that HF-P markedly outperforms Pro-Q DPS.

### 3.2. Phosphorylation Level of Proteins

The *PR* statistics [35,48] was used to evaluate quantitative changes in phosphorylation status, but in a limited region of the gel. It was assessed by the difference in spot volumes between control and treated samples. The following observations (Figure 2) on the dephosphorylation pattern at global level can be appreciated: (1) First, several spots expressed in the reference pattern exhibited less volume on identical gel positions at dephosphorylated protein profiles (e.g., spots 88, 96 and 116). This pattern could be explained by the fact that these spots are formed by a mixture of phosphorylated and unphosphorylated polypeptides, and after applying HF-P to remove phosphate moieties, the volumes are reduced proportionately. (2) Some spots were shifted to more basic positions into their p*I* and with decrease in volume (e.g., spots 105–107). During dephosphorylation, there is loss of phosphate groups on the residues of phosphotyrosine, phosphothreonine and phosphoserine [49]. This loss is replenished by neutral hydroxyl groups which induce changes on the p*I*. It is then possible to observe variable displacements towards the basic region either by slight dephosphorylation or by a higher increase in the p*I* due to numerous dephosphorylations. (3) It is also shown that the most intense and faint spots were undetected after dephosphorylation treatment with HF-P. This finding could be explained by whether the absent spots contained only phosphorylated polypeptides. Regarding this, Wu et al. [50] showed that multi-phosphorylated proteins altered the electrophoretic mobility in SDS-PAGE. This study showed a displacement of 10 kDa when five phosphate groups were eliminated from β-casein protein. However, the mass difference between β-casein/*5pβ*-casein was 400 Da when analyzed by MS (80 Da per phosphate group) [50].This discordance could be explained by the fact that the mobility of proteins in SDS-PAGE is related to the number of SDS molecules [51]. The introduction of negative charges by phosphorylation implies a fewer number of SDS molecules binding to protein due to the repulsion. Contrarily, the elimination of phosphate groups allows for the addition of more SDS molecules and consequently more displacement in SDS-PAGE [52]. Regarding our results, it could be hypothesized that the total elimination of multiple phosphate groups after dephosphorylation might imply the union of SDS molecules leading to a considerable displacement and elimination of spots from the map of 2-DE. (4) Lastly, some novel spots were discovered only in dephosphorylated patterns. These spots could come from other dephosphorylated spots that shifted to more basic positions and with lower *M*_r_ [35].

Analysis of protein phosphorylation levels was also performed using Pro-Q DPS (Table S1 and S2). In a general overview, the number of phosphorylated spots detected was statistically significantly higher when applying HF-P (152 out of 174 spots) than Pro-Q DPS (44 out of 174) method (*p*-value < 0.0001, two-tailed Fisher’s exact test) (Figure S1). Statistically significant differences for mean *PR* values between Pro-Q DPS and chemical dephosphorylation methods were evaluated by the bias-corrected percentile approach, using 95% bootstrap CIs, adjusted with the Bonferroni correction. In total, 76.4% (133 out of 174) of phosphorylated protein spots showed statistically significant differences in the mean *PR*-value (Table S1). Significant differences over phosphoprotein spots were marked and numbered in Figure 2.

Our observations revealed that most spots with a significant difference in *PR* between methods were not detectable on gel images after staining with Pro-Q DPS dye (93 out of 122 spots) (Table S1). However, undetected spots with the phosphostain does not mean that they were unphosphorylated. Low-abundant phosphoproteins were not visualized by the Pro-Q DPS stain because they are below the detection threshold. Although there are several investigations in which they stand out, the sensitivity of Pro-Q DPS bears a limit of detection as low as 4 ng per spot [19]. It is worth noting that the gels, after HF-P treatment, were stained with Sypro Ruby with a detection sensitivity of 1 ng [53]. These contrasting thresholds of detection could explain the aforementioned difference between techniques. On the other hand, although Pro-Q DPS is used to conduct a global quantitative analysis of phosphoproteins due to its direct binding to the phosphate groups of phosphoproteins [54], it must be revealed that Murray et al. [23] demonstrated some weakness regarding the detection of nonphosphorylated proteins that can cause false positives. Conversely, the Sypro Ruby stain is based on a luminescent metal chelate stain composed of ruthenium into an organic complex [55], which interacts noncovalently with proteins. Therefore, it stains all proteins independently of the number of phosphate groups. The method with HF-P was visualized with Sypro Ruby, allowing for identification of phosphorylated spots and solving the problems presented by phosphostains.

### 3.3. Efficiency Assessment of the Dephosphorylation Method with HF-P

**Figure 3:**
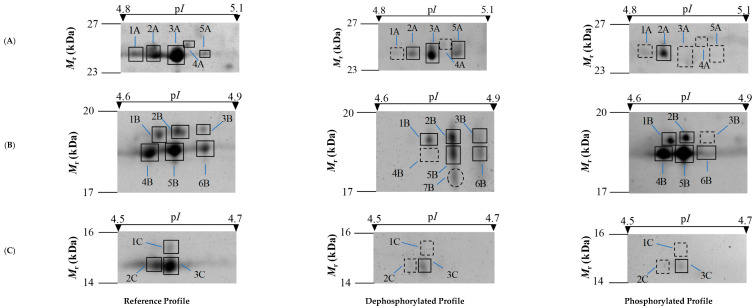
Representative 2-DE gel images of reference, dephosphorylated and phosphorylated profiles of three different zones (**A**–**C**). Reference profiles on 2-DE gels were stained with Sypro Ruby fluorescent dye. Dephosphorylated profiles on 2-DE gels were stained with Sypro Ruby from total protein extracts treated with HF-P. Phosphorylated profiles on 2-DE gels were stained with Pro-Q DPS. Spots studied are numbered. The dashed squares represent missing spots either after dephosphorylation with HF-P (dephosphorylated profile) or non-phosphorylated spots identified with Pro-Q DPS (phosphorylated profile). Dashed circles represent newly arisen spots after dephosphorylation as compared with reference patterns.

In total, three different target zones of 2-DE gels were sampled to estimate the efficiency of dephosphorylation with HF-P (Figure 3). Spots were matched by PDQuest software and analyzed by MALDI-TOF and MALDI-TOF/TOF MS and those spots not identified were checked with the literature. The results of protein identifications are shown in Table 1.

Firstly, 2-DE gel spots were studied within a selected pattern from *M*_r_ of 23 to 27 kDa and a p*I* from 4.8 to 5.1 (Figure 3A). Protein identifications in zone A revealed two different proteins: myosin light chain 1/3 (MYL1) (spots 2A and 3A) and myosin light chain 3 (MYL3, spot 4A). In this area, the rate of phosphorylation (*PR_HF-P_*) ranged from 47.9 to 100% (Table 2). Both a decrease in the volume (2A and 3A) and the disappearance of spots were observed after the treatment with HF-P (spots 1A and 4A). In contrast, spot 5A increased in volume (Table S3). This last result could be explained by the fact that spot 4A moved to a basic position after the elimination of 100% of their phosphate groups [35]. In contrast, the Pro-Q DPS method showed a lower resolution capacity, finding only the spot 2A as phosphorylated in this area. In addition, this method provided a high variability in *PR*_Pro-Q DPS_ for this spot over gel replicates, as shown by the standard error (SE) of the mean value (mean ± SE was 27.2 ± 16.6) (Table 2).

The selected pattern from 17 to 20 kDa and a p*I* from 4.6 to 4.9 that corresponds to the zone B (Figure 3B) was also examined. Protein identifications in zone B revealed two different proteins: Myosin regulatory light chain 2, ventricular/cardiac muscle isoform (MYL2, spots 1B-3B) and myosin regulatory light chain 2, fast skeletal muscle isoform (MYLPF, spots 4B-6B) (Table 1). After HF-P treatment, all spots underwent a pronounced movement from acid to basic positions, hypothesizing that these spots can be phosphorylated. Moreover, a new 7B spot arising after dephosphorylation could come from a spot with higher *M*_r_ (Figure 3B). Furthermore, Pro-Q DPS showed an ultra-saturated pattern in those spots with a higher volume (Table S3) in reference profile (spots 1B, 2B, 4B, 5B), approximating the *PR*_Pro-Q DPS_ to 100% (Table 2). On the contrary, higher efficiency was observed with the chemical method, obtaining very precise values of *PR*_HF-P_ (Table 2). Note that the phosphostain was unable to detect phosphate groups in the spot 3B of scarce volume (Table S3) due to its low sensibility. In contrast, a *PR*_HF-P_ value of 22.6% was detected in this spot with the HF-P method.

In addition, the area C that included the selected pattern from *M*r 14 kDa to 16 kDa and p*I* from 4.5 to 4.7 (Figure 3C) was studied. Myosin regulatory light chain 2, ventricular/cardiac muscle isoform (MYL2, spot 1C) and MYL1 protein (MYL1, spots 2C and 3C) were identified in this area. Dephosphorylation method showed that the three spots were phosphorylated, while Pro-Q DPS was able to detect only the spot 3C due to its high volume (Table 2; Table S3). It is worth noting that the HF-P method has higher accuracy and sensibility than Pro-Q DPS. Moreover, the phosphostain can detect only those spots with a higher amount of phosphoprotein. In contrast, HF-P enables the identification of phosphorylated spots with low volume in total.

It is well-recognized that the muscle myosin light chains (MYL1, MYL3) and regulatory light chain 2 isoforms (MYL2 and MYLPF) are phosphorylated. In previous research, MYL1 showed some phosphorylated isoforms on 2-DE gels staining with ProQ-DPS [59]. However, more phosphorylated isoforms were discovered with HF-P, which allows a better understanding about the importance of the modulatory role of MYL1 in muscle contraction [57]. On the other hand, the phosphorylation of protein MYL2, which was identified in section B of Figure 3, is related to the rigor mortis progress and has an important regulatory role in striated muscle contraction [3]. Regarding MYL3, few papers have reported phosphorylated isoforms using ProQ-DPS. However, Zhang et al. [60] discovered phosphorylation sites in the ovine homologue. This evidence emphasizes the efficiency of HF-P at detecting phosphorylated proteins. Lastly, phosphorylation of MYLPF was detected with both methods. It is an important phosphoprotein with an important role in meat tenderness [3,15]. Overall, our study shows a higher resolution power of HF-P for analyzing phosphorylated proteins, enabling the obtention of more precise *PR*-values.

### 3.4. Importance of HF-P on the Study of Phosphoproteome at Global Level

Several techniques have been developed in recent years to detect phosphorylated proteins, including immunoblotting [5], radioactive labelling [61], phosphostain method [21], mass spectrometry [62], phos-tag SDS-PAGE technique [63]; electrochemical assay [64], photoluminescence [65] or colorimetric detection [64], among others. One of the most recently used techniques is the detection of phosphoproteins with antibodies [5]; however, it has been surpassed by MS. Despite the breakthrough in the field of MS, phosphorylation remains a challenge due to the low PTM stoichiometry, poor quality MS/MS spectra and the need to develop improvements in bioinformatic analyses associated with a large amount of data generated which increases false discovery rates [4]. Therefore, researchers have noticed the strength of 2-DE-MS, which provides the possibility to identify and detect protein modifications in those cases where no efficient affinity enrichment and MS-based methods exist [9,66].

The capabilities of 2-DE-MS have increased substantially in this study by obtaining 2-DE-based global phosphoproteome map using chemical dephosphorylation with HF-P. Note that this method was previously applied only in targeted proteomic studies aimed to analyze isoforms of a reduced number of proteins [34,35,36,37]. HF-P was found to be more efficient than Pro-Q for multiplex analysis of the phosphoproteome. First, HF-P requires only a unique stain (SYPRO) to evaluate the spots, in contrast with the phosphostain method (Pro-Q DPS and SYPRO), which can avoid the possible variance when comparing spots with different dyes at the bioinformatic level [67]. Second, HF-P coupled to 2-DE improves the separation of protein variants with different net electric charge and different molecular mass, resulting from dephosphorylation. Third, HF-P is a more sensitive method being able to detect low-abundant phosphoproteins. For all these reasons, the present study develops one of the strengths of 2-DE allowing for the analysis of entire proteins and their variant forms.

### 3.5. Importance of HF-P in the Meat Industry

This technique can be applied in the food industry, and more specifically in meat commerce, to increase knowledge of the phosphorolytic processes which underlie the conversion of muscle to meat that would allow a better understanding of variations in beef quality (such as in meat tenderness, color stability, shear force, etc.) [3,59,68]. Currently, protein biomarkers are key tools in the food industry to investigate food origin, composition, additives, breed identification or quality [38,69], as well as to develop safe food products through their authentication, food toxicity or product traceability [39,70]. In particular, a recent review by Afzaal et al. [40] shows the importance of proteomics in the authentication of food and in particular the detection of adulteration in meat. Phosphoproteomic approaches can be used to search for biomarkers of meat quality, as it allows the evaluation of spatio-temporal plasticity variations of the proteome. For instance, the study of phosphoproteome was used to evaluate variation during postmortem meat processing [71], variations in meat tenderness [72], changes associated to different ultimate pH [68], to evaluate the differences between fast and slow growth in broilers [73], sarcoplasmic protein variations in pigs during the four sessions [74], differences between goat muscle of different quality [75] or be used as poultry goose meat age markers [76], among others. With regard to this, the application of the HF-P technique through high-resolution 2-DE profiles in meat samples allows for the refinement of methods to evaluate the amount of phosphoprotein in meat proteome and to unravel the molecular mechanisms underlying the biological variability associated with meat quality.

## 4. Conclusions

The results lead us to conclude that the method of chemical dephosphorylation with FH-P is considerably more efficient than the method of phosphostain with Pro-Q DPS to unraveling the phosphoproteome, both qualitatively and quantitatively. Thus, we found that 2-DE phosphoproteome profiles resulting from bovine meat samples treated with FH-P were able to detect a significantly (*p*-value < 0.05) higher number of phosphoproteins (three-fold increase) than Pro-Q DPS. In addition, FH-P exhibited a higher relative sensitivity to detect isoforms with different levels of phosphorylation and low-abundant phosphoproteins. It follows that FP-H can provide a wider phosphoproteome coverage than Pro-Q DPS. Furthermore, this method is compatible with MS technologies providing a powerful tool for the multiplex identification and quantification of phosphoproteins. Overall, the HF-P method provides a new technology to decipher the phosphoproteome that adds to the existing 2-DE-based proteomic methodologies. It can be particularly useful to gain a deeper understanding of phosphoproteome in food sciences. More specifically, the HF-P method can be applied in follow-up studies to assess phosphoproteome changes underlying muscle-to-meat conversion and the identification of putative biomarkers linked to high-quality meats.

## Figures and Tables

**Table 1:** Identification of protein spots selected from three representative 2-DE gel by MALDI-TOF, MALDI-TOF/TOF MS or contrasted with the literature in those unidentified spots by MS.

Spot Code ^1^	Protein ^2^	Abbrev.	Accession No. (Uniprot)	Mascot Score	Sequence Cov. (%)	No. of Matched Peptides	p*I* Th/ Obs ^3^	*M*r Th/ Obs (kDa) ^3^	Method of Identification
1A	--	--	--	--	--	--	--/	--/	
2A	Myosin light chain 1/3, skeletal muscle isoform	MYL1	A0JNJ5	170	41	8	4.96/4.87	21.0/24.8	MALDI-TOF and MALDI-TOF/TOF
3A	Myosin light chain 1/3, skeletal muscle isoform	MYL1	A0JNJ5	--	45	14	4.96/4.95	21.0/24.8	[56]
4A	Myosin light chain 3	MYL3	P85100	--	43	4	5.00/4.99	21.9/25.2	[41]
5A	--	--	--	--	--	--	--/	--/	
1B	Myosin regulatory light chain 2, ventricular/cardiac muscle isoform.	MYL2	Q3SZE5	200	63	12	4.86/4.70	18.9/18.8	MALDI-TOF and MALDI-TOF/TOF
2B	Myosin regulatory light chain 2, ventricular/cardiac muscle isoform.	MYL2	Q3SZE5	166	60	11	4.86/4.75	18.9/18.8	MALDI-TOF and MALDI-TOF/TOF
3B	Myosin regulatory light chain 2, ventricular/cardiac muscle isoform.	MYL2	Q3SZE5	166	60	13	4.86/4.80	18.0/18.8	[56,57]
4B	Myosin regulatory light chain 2, skeletal muscle isoform	MYLPF	Q0P571	134	20	3	4.91/4.68	19.1/18.5	MALDI-TOF and MALDI-TOF/TOF
5B	Myosin regulatory light chain 2, fast skeletal muscle isoform	MYLPF	Q0P571	126	20	5	4.91/4.73	19.1/18.5	MALDI-TOF and MALDI-TOF/TOF
6B	Myosin regulatory light chain 2, fast skeletal muscle isoform	MYLPF	Q0P571	126	80	22	4.91/4.81	19.1/18.5	[57,58]
7B	--	--	--	--	--	--	--	--	
1C	Myosin regulatory light chain 2, ventricular/cardiac muscle isoform	MYL2	Q3SZE5	221	23	9	4.86/4.60	18.9/15.5	[41]
2C	MYL1 protein	MYL1	Q08E10	239	52	9	4.73/4.57	19.7/15.0	MALDI-TOF and MALDI-TOF/TOF
3C	MYL1 protein	MYL1	Q08E10	97	32	5	4.73/4.60	19.7/15.0	MALDI-TOF and MALDI-TOF/TOF

^1^ Spot position is shown in Figure 3; ^2^ Identification of proteins matched to *B. taurus* protein databases; ^3^ Theoretical (Th) p*I* and *M*r were obtained from UniProtKB/Swiss-Prot databases.

**Table 2:** Mean (±SE) of phosphorylation rate (*PR*) and its 95% CI for spots selected from three representative 2-DE gel zones obtained by HF-P and Pro-Q DPS methods.

Spot Code ^1^	*PR*				*p*-Value ^4^	*PR* Representation ^5^
	HF-P		Pro-Q DPS			
	Mean (±SE) ^2^	95% Bootstrap CI (CL, CU) ^3^	Mean (±SE) ^2^	95% Bootstrap CI (CL, CU) ^3^		
1A	100 ± 0.0	100, 100	0.0 ± 0.0	0.0, 0.0	<0.05	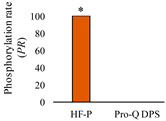
2A	87.4 ± 3.7	83.2, 94.2	27.2 ± 16.6	8.9, 59.7	<0.05	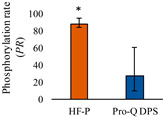
3A	47.9 ± 8.3	38.5, 63.2	0.0 ± 0.0	0.0, 0.0	<0.05	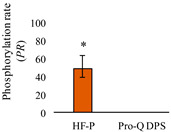
4A	100 ± 0.0	100, 100	0.0 ± 0.0	0.0, 0.0	<0.05	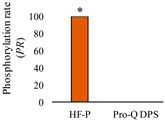
5A	N/A	N/A	0.0 ± 0.0	0.0, 0.0	--	--
1B	59.0 ± 2.1	56.5, 62.8	100 ± 0.0	100, 100	<0.05	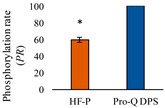
2B	32.5 ± 5.0	36.5, 38.5	100 ± 0.0	100, 100	<0.05	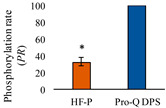
3B	22.6 ± 9.4	4.7, 36.8	0 ± 0.0	0, 0	<0.05	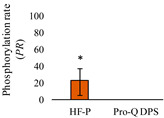
4B	100 ± 0.0	100, 100	100 ± 0.0	100, 100	ns	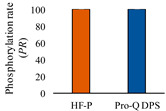
5B	69.6 ± 5.4	63.3, 79.5	100 ± 0.0	100, 100	<0.05	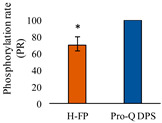
6B	63.7 ± 21.4	37.5, 89.9	51.8 ± 17.7	34.0, 87.3	ns	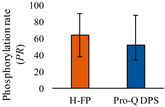
7B	N/A	N/A	--	--	--	--
1C	100 ± 0.0	100, 100	0.0 ± 0.0	0.0, 0.0	<0.05	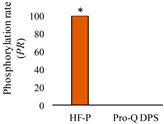
2C	100 ± 0.0	100, 100	0.0 ± 0.0	0.0, 0.0	<0.05	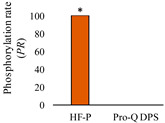
3C	88.8 ± 3.9	81.4, 94.9	5.5 ± 5.3	0.1, 16.0	< 0.05	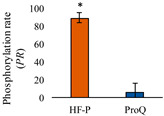

^1^ Gel position of numbered spots is shown in Figure 3; ^2^ Data are means of *PR* ± standard error from three biological replicates; ^3^ CI, confidence interval; CL, lower bound; CU, upper bound. The 95% bootstrap CIs were acquired by the bias-corrected percentile method and corrected by the Bonferroni method; ^4^ *p*-value < 0.05: statistically significant difference in mean *PR* between methods; ns: no statistically significant difference (*p* > 0.05); ^5^ Asterisk indicates a significant difference (*p*-value < 0.05) in mean *PR* between methods; N/A = not applicable, spot with more volume in treated samples than untreated with HF-P.

## Data Availability

Data are contained within the article or Supplementary Materials.

## Supplementary Materials

The following supporting information can be downloaded at: https://www.mdpi.com/article/10.3390/foods11193119/s1, Table S1: Mean volume of *PR* (±SE) and 95% CI interval for 2-DE spots with statistically significant differential phosphorylation rate (*PR*) between HF-P and Pro-Q DPS methods to evaluate phosphorylation from LT muscle meat of Rubia Gallega (RG). Table S2: Phosphorylation rate (*PR*) of each replicate and mean (±SE) for 2-DE spots with statistically significant different *PR* between two methods (HF-P and Pro-Q DPS) to evaluate phosphorylation in meat samples from LT muscle. Table S3: Volume of spots for untreated protein samples, dephosphorylated spots with HF-P and phosphorylated spots visualized with Pro-Q DPS were assessed by PDQuest software. Figure S1: Number of phosphorylated protein spots obtained with HF-P and Pro-Q DPS method (* *p* < 0.0001, Fisher’s exact test).
