# Supplementary material for: Phosphoproteome Analysis Using Two-Dimensional Electrophoresis Coupled with Chemical Dephosphorylation
Source: Foods. 2022 Oct 7;11(19):3119. doi: 10.3390/foods11193119 (PMC9562008; doi:10.3390/foods11193119)
Supplement: Supplementary file 1 [file foods-11-03119-s001.zip › foods-1856572-supplementary.pdf]

Supplementary Material

Phosphoproteome Analysis Using Two-Dimensional Electrophoresis Coupled with Chemical Dephosphorylation

Raquel Rodríguez-Vázquez *, Daniel Mouzo and Carlos Zapata

Supplementary Tables S1–3

Supplementary Figure S1

Table S1

Mean volume of *PR* (±SE) and 95% CI interval for 2-DE spots with statistically significant differential phosphorylation rate (*PR*) between HF-P and Pro-Q DPS methods to evaluate phosphorylation from LT muscle meat of Rubia Gallega (RG).

	$PR_{HF\text{-}P}$		$PR_{ProQ\text{-}DPS}$		
**Spot code**[1]	**Volume ± SE**[2]	**95% bootstrap CI (CL, CU)**[3]	**Volume ± SE**[2]	**95% bootstrap CI (CL, CU)**[3]	***P*-value**[4]
1	100 ± 0.0	100, 100	0	0, 0	<0.05
2	100 ± 0.0	100, 100	0	0, 0	<0.05
3	100 ± 0.0	100, 100	0	0, 0	<0.05
4	100 ± 0.0	100, 100	0	0, 0	<0.05
5	52 ± 15.0	33.2, 69.9	0	0, 0	<0.05
6	47 ± 13.0	22.5, 68.8	0	0, 0	<0.05
7	100 ± 0.0	100, 100	0	0, 0	<0.05
8	100 ± 0.0	100, 100	0	0, 0	<0.05

9	100 ± 0.0	100, 100	0	0, 0	<0.05
10	100 ± 0.0	100, 100	0	0, 0	<0.05
11	100 ± 0.0	100, 100	13 ± 3.0	0.07, 0.18	<0.05
12	100 ± 0.0	100, 100	10 ± 3.0	5.2, 14.9	<0.05
13	100 ± 0.0	100, 100	0	0, 0	<0.05
14	100 ± 0.0	100, 100	0	0, 0	<0.05
15	100 ± 0.0	100, 100	0	0, 0	<0.05
16	100 ± 0.0	100, 100	0	0, 0	<0.05
17	100 ± 0.0	100, 100	0	0, 0	<0.05
18	100 ± 0.0	100, 100	71 ± 10	60.0, 89.6	<0.05
19	100 ± 0.0	100, 100	48 ± 15	18.0, 69.0	<0.05
20	100 ± 0.0	100, 100	51 ± 12	37.8, 73.0	<0.05
21	100 ± 0.0	100, 100	0	0, 0	<0.05
22	100 ± 0.0	100, 100	0	0, 0	<0.05

23	100 ± 0.0	100, 100	0	0, 0	<0.05
24	100 ± 0.0	100, 100	0	0, 0	<0.05
25	100 ± 0.0	100, 100	0	0, 0	<0.05
26	100 ± 0.0	100, 100	0	0, 0	<0.05
27	100 ± 0.0	100, 100	0	0, 0	<0.05
28	100 ± 0.0	100, 100	0	0, 0	<0.05
29	100 ± 0.0	100, 100	0	0, 0	<0.05
30	100 ± 0.0	100, 100	0	0, 0	<0.05
31	100 ± 0.0	100, 100	0	0, 0	<0.05
32	23 ± 11.0	15, 38.0	0	0, 0	<0.05
33	100 ± 0.0	100, 100	0	0, 0	<0.05
34	100 ± 0.0	100, 100	0	0, 0	<0.05
35	100 ± 0.0	100, 100	0	0, 0	<0.05
36	100 ± 0.0	100, 100	0	0, 0	<0.05

37	100 ± 0.0	100, 100	0	0, 0	<0.05
38	100 ± 0.0	100, 100	0	0, 0	<0.05
39	100 ± 0.0	100, 100	69 ± 5.0	58.0, 76.3	<0.05
40	100 ± 0.0	100, 100	61 ± 13	34.2, 77.4	<0.05
41	100 ± 0.0	100, 100	0	0, 0	<0.05
42	100 ± 0.0	100, 100	0	0, 0	<0.05
43	100 ± 0.0	100, 100	84 ± 11	61.5, 96.6	<0.05
44	100 ± 0.0	100, 100	88 ± 3.0	82.3, 94.3	<0.05
45	100 ± 0.0	100, 100	0	0, 0	<0.05
46	100 ± 0.0	100, 100	0	0, 0	<0.05
47	100 ± 0.0	100, 100	0	0, 0	<0.05
48	100 ± 0.0	100, 100	72 ± 15	46.5, 97.6	<0.05
49	100 ± 0.0	100, 100	86 ± 4.7	76.5, 91.9	<0.05
50	100 ± 0.0	100, 100	75 ± 8.0	58.2, 87.1	<0.05

51	100 ± 0.0	100, 100	66 ± 21	27.0, 99.6	<0.05
52	100 ± 0.0	100, 100	0	0, 0	<0.05
53	100 ± 0.0	100, 100	0	0, 0	<0.05
54	100 ± 0.0	100, 100	0	0, 0	<0.05
55	100 ± 0.0	100, 100	80 ± 7.0	72.7, 92.8	<0.05
56	100 ± 0.0	100, 100	86 ± 8.0	72.7, 98.4	<0.05
57	100 ± 0.0	100, 100	0	0, 0	<0.05
58	100 ± 0.0	100, 100	0	0, 0	<0.05
59	100 ± 0.0	100, 100	21 ± 9.0	10.3, 39.0	<0.05
60	100 ± 0.0	100, 100	78 ± 7.0	70.6, 91.8	<0.05
61	15 ± 6.0	3.0, 26	0	0, 0	<0.05
62	100 ± 0.0	100, 100	0	0, 0	<0.05
63	100 ± 0.0	100, 100	0	0, 0	<0.05
64	100 ± 0.0	100, 100	0	0, 0	<0.05

65	100 ± 0.0	100, 100	0	0, 0	<0.05
66	100 ± 0.0	100, 100	0	0, 0	<0.05
67	27 ± 8.0	12.0, 38.0	0	0, 0	<0.05
68	100 ± 0.0	100, 100	0	0, 0	<0.05
69	100 ± 0.0	100, 100	0	0, 0	<0.05
70	45.2 ± 19.0	23.5, 8	0	0, 0	<0.05
71	100 ± 0.0	100, 100	0	0, 0	<0.05
72	100 ± 0.0	100, 100	0	0, 0	<0.05
73	35 ± 14	19.0, 61.0	0	0, 0	<0.05
74	100 ± 0.0	100, 100	0	0, 0	<0.05
75	100 ± 0.0	100, 100	0	0, 0	<0.05
76	100 ± 0.0	100, 100	0	0, 0	<0.05
77	100 ± 0.0	100, 100	0	0, 0	<0.05
78	100 ± 0.0	100, 100	0	0, 0	<0.05

79	100 ± 0.0	100, 100	0	0, 0	<0.05
80	100 ± 0.0	100, 100	51 ± 15	24.5, 75.4	<0.05
81	100 ± 0.0	100, 100	73 ± 10	42.6, 98.1	<0.05
82	17 ± 12	5.2, 40.7	0	0, 0	<0.05
83	52 ± 11	38.6, 64.6	0	0, 0	<0.05
84	100 ± 0.0	100, 100	0	0, 0	<0.05
85	100 ± 0.0	100, 100	72 ± 5	66.8, 81.0	<0.05
86	100 ± 0.0	100, 100	85 ± 7	71.4, 94.7	<0.05
87	100 ± 0.0	100, 100	81 ± 3	78.0, 87.7	<0.05
88	9 ± 2	6, 13	0	0, 0	<0.05
89	100 ± 0.0	100, 100	0	0, 0	<0.05
90	100 ± 0.0	100, 100	0	0, 0	<0.05
91	100 ± 0.0	100, 100	0	0, 0	<0.05
92	87.4 ± 3.7	83.2, 94.2	27.2 ± 16.6	8.9, 59.7	<0.05

93	47.9 ± 8.3	38.5, 63.2	0	0, 0	<0.05
94	100 ± 0.0	100, 100	0	0, 0	<0.05
95	100 ± 0.0	100, 100	0	0, 0	<0.05
96	53 ± 18	32.1, 86.2	0	0, 0	<0.05
97	100 ± 0.0	100, 100	0	0, 0	<0.05
98	100 ± 0.0	100, 100	0	0, 0	<0.05
99	100 ± 0.0	100, 100	0	0, 0	<0.05
100	100 ± 0.0	100, 100	0	0, 0	<0.05
101	100 ± 0.0	100, 100	0	0, 0	<0.05
102	100 ± 0.0	100, 100	0	0, 0	<0.05
103	100 ± 0.0	100, 100	0	0, 0	<0.05
104	16 ± 3.0	13.0, 19.0	0	0, 0	<0.05
105	59 ± 2.1	56.5, 62.8	100 ± 0.0	100, 100	<0.05
106	32.5 ± 5.0	36.5, 38.5	100 ± 0.0	100, 100	<0.05

107	22.6 ± 9.4	4.7, 36.8	0	0, 0	<0.05
108	100 ± 0.0	100, 100	51 ± 13	37.5, 76.1	<0.05
109	69.6 ± 5.4	63.3, 79.5	100 ± 0.0	100, 100	<0.05
110	100 ± 0.0	100, 100	88 ± 14	47.1, 64.8	<0.05
111	100 ± 0.0	100, 100	0	0, 0	<0.05
112	100 ± 0.0	100, 100	0	0, 0	<0.05
113	100 ± 0.0	100, 100	0	0, 0	<0.05
114	100 ± 0.0	100, 100	0	0, 0	<0.05
115	88 ± 3.9	81.4, 94.9	5.5 ± 5.3	0.1,16.0	<0.05
116	90 ± 3	84, 95	0	0, 0	<0.05
117	100 ± 0.0	100, 100	0	0, 0	<0.05
118	35 ± 14	30, 39	0	0, 0	<0.05
119	100 ± 0.0	100, 100	0	0, 0	<0.05
120	100 ± 0.0	100, 100	0	0, 0	<0.05

121	100 ± 0.0	100, 100	0	0, 0	<0.05
122	100 ± 0.0	100, 100	0	0, 0	<0.05
1′	N/A	--			<0.05
2′	N/A	--			<0.05
3′	N/A	--			<0.05
4′	N/A	--			<0.05
5′	N/A	--			<0.05
6′	N/A	--			<0.05
7′	N/A	--			<0.05
8′	N/A	--			<0.05
9′	N/A	--			<0.05
10′	N/A	--			<0.05
11′	N/A	--			<0.05

[1]Gel position of marked spots is shown in Fig. 2.

[2]Mean values and standard error of *PR*.

[3]Non-parametric bootstrap CIs (CL, lower bound; CU, upper bound) determined by the bias corrected percentile method.

[4]Statistically significant differences ($P < 0.05$) in phosphorylation rate between methods.

N/A: More volume in untreated than treated spot with HF-P method

Table S2

Phosphorylation rate (*PR*) of each replicate and mean (± SE) for 2-DE spots with statistically significant different *PR* between two methods (HF-P and Pro-Q DPS) to evaluate phosphorylation in meat samples from LT muscle.

	PR_{HF-P}				$PR_{Pro-Q\ DPS}$			
**Spot code[1]**	**Replicate 1**	**Replicate 2**	**Replicate 3**	**Mean ± SE[2]**	**Replicate 1**	**Replicate 2**	**Replicate 3**	**Mean ± SE[2]**
1	100	--	100	100 ± 0.0	0	0	0	0.0 ± 0.0
2	100	100	100	100 ± 0.0	0	0	0	0.0 ± 0.0
3	--	100	100	100 ± 0.0	0	0	0	0.0 ± 0.0
4	100	100	100	100 ± 0.0	0	0	0	0.0 ± 0.0
5	69.9	33.3	--	52.0 ± 15.0	0	0	0	0.0 ± 0.0
6	51.9	22.5	66.8	47 ± 13.0	0	0	0	0.0 ± 0.0
7	100	100	100	100 ± 0.0	0	0	0	0.0 ± 0.0
8	100	100	100	100 ± 0.0	0	0	0	0.0 ± 0.0
9	100	100	100	100 ± 0.0	0	0	0	0.0 ± 0.0

10	100	100	100	100 ± 0.0	0	0	0	0.0 ± 0.0
11	100	100	100	100 ± 0.0	14.56	7.02	18.38	13 ± 3.0
12	100	100	100	100 ± 0.0	14.93	5.21	10.59	10 ± 3.0
13	100	100	100	100 ± 0.0	0	0	0	0.0 ± 0.0
14	100	100	100	100 ± 0.0	0	0	0	0.0 ± 0.0
15	100	100	100	100 ± 0.0	0	0	0	0.0 ± 0.0
16	100	100	100	100 ± 0.0	0	0	0	0.0 ± 0.0
17	100	100	100	100 ± 0.0	0	0	0	0.0 ± 0.0
18	100	100	100	100 ± 0.0	57.44	65.11	89.63	71 ± 10
19	100	100	100	100 ± 0.0	17.98	69.04	57.49	48 ± 15
20	100	100	100	100 ± 0.0	47.09	33.18	73.03	51 ± 12
21	100	100	100	100 ± 0.0	0	0	0	0.0 ± 0.0
22	100	100	100	100 ± 0.0	0	0	0	0.0 ± 0.0
23	100	100	100	100 ± 0.0	0	0	0	0.0 ± 0.0
24	100	100	100	100 ± 0.0	0	0	0	0.0 ± 0.0

25	100	100	100	100 ± 0.0	0	0	0	0.0 ± 0.0
26	100	100	100	100 ± 0.0	0	0	0	0.0 ± 0.0
27	100	100	100	100 ± 0.0	0	0	0	0.0 ± 0.0
28	100	100	100	100 ± 0.0	0	0	0	0.0 ± 0.0
29	100	100	100	100 ± 0.0	0	0	0	0.0 ± 0.0
30	100	100	100	100 ± 0.0	0	0	0	0.0 ± 0.0
31	100	100	100	100 ± 0.0	0	0	0	0.0 ± 0.0
32	38.3	1.5	29.3	23 ± 11.0	0	0	0	0.0 ± 0.0
33	100	100	100	100 ± 0.0	0	0	0	0.0 ± 0.0
34	100	100	100	100 ± 0.0	0	0	0	0.0 ± 0.0
35	100	100	100	100 ± 0.0	0	0	0	0.0 ± 0.0
36	100	100	100	100 ± 0.0	0	0	0	0.0 ± 0.0
37	100	100	100	100 ± 0.0	0	0	0	0.0 ± 0.0
38	100	100	100	100 ± 0.0	0	0	0	0.0 ± 0.0
39	100	100	100	100 ± 0.0	76.29	58.03	72.04	69 ± 5.0

40	100	100	100	100 ± 0.0	34.19	77.43	70.54	61 ± 13
41	100	100	100	100 ± 0.0	0	0	0	0.0 ± 0.0
42	100	100	100	100 ± 0.0	0	0	0	0.0 ± 0.0
43	100	100	100	100 ± 0.0	96.63	61.54	91.41	84 ± 11
44	100	100	100	100 ± 0.0	94.33	82.33	89.10	88 ± 3.0
45	100	100	100	100 ± 0.0	0	0	0	0.0 ± 0.0
46	100	100	100	100 ± 0.0	0	0	0	0.0 ± 0.0
47	100	100	100	100 ± 0.0	0	0	0	0.0 ± 0.0
48	100	100	100	100 ± 0.0	46.52	73.14	97.58	72 ± 15
49	100	100	100	100 ± 0.0	89.07	76.55	91.90	86 ± 5.0
50	100	100	100	100 ± 0.0	87.13	80.49	58.17	75 ± 8.0
51	100	100	100	100 ± 0.0	99.65	26.97	71.92	66 ± 21
52	100	100	100	100 ± 0.0	0	0	0	0.0 ± 0.0
53	100	100	100	100 ± 0.0	0	0	0	0.0 ± 0.0
54	100	100	100	100 ± 0.0	0	0	0	0.0 ± 0.0

55	100	100	100	100 ± 0.0	92.80	75.21	71.59	80 ± 7.0
56	100	100	100	100 ± 0.0	89.0	72.0	98.0	86 ± 8.0
57	100	100	100	100 ± 0.0	0	0	0	0.0 ± 0.0
58	100	100	100	100 ± 0.0	0	0	0	0.0 ± 0.0
59	100	100	100	100 ± 0.0	7.36	39.2	16.18	21 ± 9.0
60	100	100	100	100 ± 0.0	91.83	71.18	70.29	78 ± 7.0
61	26.2	3.4	15.2	15 ± 6.0	0	0	0	0.0 ± 0.0
62	100	100	100	100 ± 0.0	0	0	0	0.0 ± 0.0
63	100	100	100	100 ± 0.0	0	0	0	0.0 ± 0.0
64	100	100	100	100 ± 0.0	0	0	0	0.0 ± 0.0
65	100	100	100	100 ± 0.0	0	0	0	0.0 ± 0.0
66	100	100	100	100 ± 0.0	0	0	0	0.0 ± 0.0
67	12.2	31.7	38.0	27 ± 8.0	0	0	0	0.0 ± 0.0
68	100	100	100	100 ± 0.0	0	0	0	0.0 ± 0.0
69	100	100	100	100 ± 0.0	0	0	0	0.0 ± 0.0

70	14.4	41.6	79.8	45.2 ± 19.0	0	0	0	0.0 ± 0.0
71	100	100	--	100 ± 0.0	0	0	--	0.0 ± 0.0
72	100	100	100	100 ± 0.0	0	0	0	0.0 ± 0.0
73	12.0	33.5	60.7	35 ± 14	0	0	0	0.0 ± 0.0
74	100	100	100	100 ± 0.0	0	0	0	0.0 ± 0.0
75	100	100	--	100 ± 0.0	0	0	--	0.0 ± 0.0
76	100	100	100	100 ± 0.0	0	0	0	0.0 ± 0.0
77	100	100	100	100 ± 0.0	0	0	0	0.0 ± 0.0
78	100	100	100	100 ± 0.0	0	0	0	0.0 ± 0.0
79	100	100	100	100 ± 0.0	0	0	0	0.0 ± 0.0
80	100	100	100	100 ± 0.0	24.56	53.10	75.39	51 ± 15
81	100	100	--	100 ± 0.0	42.59	79.20	98.13	73 ± 16
82	5.2	5.2	40.7	17 ± 12	0	0	0	0.0 ± 0.0
83	38.6	--	64.7	52 ± 11	0	--	0	0.0 ± 0.0
84	100	100	--	100 ± 0.0	0	0	--	0.0 ± 0.0

85	100	100	100	100 ± 0.0	69.89	65.28	80.98	72 ± 5
86	100	100	100	100 ± 0.0	71.38	90.06	94.67	85 ± 7
87	100	100	100	100 ± 0.0	77.02	87.67	79.97	81 ± 3
88	13.4	7.6	5.3	9 ± 2	0	0	0	0.0 ± 0.0
89	100	--	100	100 ± 0.0	0	--	0	0.0 ± 0.0
90	100	100	100	100 ± 0.0	0	0	0	0.0 ± 0.0
91	100	100	100	100 ± 0.0	0	0	0	0.0 ± 0.0
92	81.5	94.2	86.6	87.4 ± 3.7	4.99	16.85	59.74	27.2 ± 16.6
93	34.8	45.8	63.3	47.9 ± 8.3	0	0	0	0.0 ± 0.0
94	100	100	100	100 ± 0.0	0	0	0	0.0 ± 0.0
95	100	100	100	100 ± 0.0	0	0	0	0.0 ± 0.0
96	24.1	48.0	86.2	53 ± 18	0	0	0	0.0 ± 0.0
97	100	--	100	100 ± 0.0	0	--	0	0.0 ± 0.0
98	100	100	100	100 ± 0.0	0	0	0	0.0 ± 0.0
99	100	100	100	100 ± 0.0	0	0	0	0.0 ± 0.0

100	100	100	100	100 ± 0.0	0	0	0	0.0 ± 0.0
101	100	100	100	100 ± 0.0	0	0	0	0.0 ± 0.0
102	--	100	100	100 ± 0.0	--	0	0	0.0 ± 0.0
103	100	100	100	100 ± 0.0	0	0	0	0.0 ± 0.0
104	--	19.4	13.0	16 ± 3.0	--	0	0	0.0 ± 0.0
105	63.0	58.8	55.5	59 ± 2.1	100	100	100	100 ± 0.0
106	38.5	22.5	36.5	32.5 ± 5.0	100	100	100	100 ± 0.0
107	4.7	36.8	26.6	22.6 ± 9.4	0	0	0	0.0 ± 0.0
108	100	100	100	100 ± 0.0	34.93	76.15	42.67	51 ± 13
109	60.8	68.5	79.5	69.6 ± 5.4	100	100	100	100 ± 0.0
110	100	--	100	100 ± 0.0	100	--	70.38	88 ± 14
111	100	100	100	100 ± 0.0	0	0	0	0.0 ± 0.0
112	--	100	100	100 ± 0.0	--	0	0	0.0 ± 0.0
113	100	100	100	100 ± 0.0	0	0	0	0.0 ± 0.0
114	100	100	100	100 ± 0.0	0	0	0	0.0 ± 0.0

115	94.91	81.38	90.06	88 ± 3.9	0	16.03	0.42	5.5 ± 5.3
116	94.9	83.9	90.0	90 ± 3	0	0	0	0.0 ± 0.0
117	100	100	100	100 ± 0.0	0	0	0	0.0 ± 0.0
118	--	30.0	39.2	35 ± 14	--	0	0	0.0 ± 0.0
119	100	100	100	100 ± 0.0	0	0	0	0.0 ± 0.0
120	100	100	100	100 ± 0.0	0	0	0	0.0 ± 0.0
121	100	100	100	100 ± 0.0	0	0	0	0.0 ± 0.0
122	100	100	100	100 ± 0.0	0	0	0	0.0 ± 0.0
1′	N/A	N/A	N/A					
2′	N/A	N/A	N/A					
3′	N/A	N/A	N/A					
4′	N/A	N/A	N/A					
5′	N/A	N/A	N/A					
6′	N/A	N/A	N/A					
7′	N/A	N/A	N/A					

8′	N/A	N/A	N/A
9′	N/A	N/A	N/A
10′	N/A	N/A	N/A
11′	N/A	N/A	N/A

[1] Gel positions of marked spots is shown in Figure 2

[2] Mean values and standard error of *PR*.

N/A: More volume in untreated than treated spot with HF-P method

Table S3

Volume of spots for untreated protein samples, dephosphorylated spots with HF-P and phosphorylated spots visualized with Pro-Q DPS were assessed by PDQuest software.

	Volume/Total protein[2]				Volume/ HF-P treatment[2]				Volume/ Pro-Q DPS[2]			
**Spot code[1]**	**Repl 1**	**Repl 2**	**Repl 3**	**Mean ± SE[3]**	**Repl 1**	**Repl 2**	**Repl 3**	**Mean ± SE[3]**	**Repl 1**	**Repl 2**	**Repl 3**	**Mean ± SE[3]**
1A	3561	1387	2737	2562 ± 634	0	0	0	0 ± 0	0	0	0	0 ± 0
2A	12396	7724.2	19575	13232 ± 3446	2295	446	2616	1786 ± 676	619	1301	11694	4538 ± 3583
3A	9611	14224	18967	14267 ± 2701	6266	7706	6966	6979 ± 416	0	0	0	0 ± 0
4A	1136	8001	--	4568 ± 2803	0	0	0	0 ± 0	0	0	0	0 ± 0
5A	2130	2393	1451	1991 ± 281	5584	7758	4382	5908 ± 988	0	0	0	0 ± 0

1B	6284	3904	5587	5258 ± 706	2337	1607	2487	2144 ± 272	14700	9875	10748	11774 ± 1484
2B	7564	5482	9379	7475 ± 1126	4651	4247	5955	4951 ± 515	14352	9697	9554	11201 ± 1576
3B	2653	3816	4182	3550 ± 461	2529	2413	3070	2671 ± 202	0	0	0	0 ± 0
4B	23123	17493	10743	17120 ± 3579	0	0	0	0 ± 0	37437	48448	48497	44794 ± 3678
5B	18864	20171	29351	22795 ± 3299	7401	6360	6008	6590 ± 418	63043	50724	66758	60175 ± 4845
6B	2089	4847	18832	8589 ± 5183	4234	3029	1905	3056 ± 672	705	4232	6496	3811 ± 1685
7B	--	--	--	--	3429	4671	3380	3826 ± 422	--	--	--	--
1C	1657	1275	4330	2421 ± 961	0	0	0	0 ± 0	0	0	0	0 ± 0
2C	2962	7239	7749	5983 ± 1518	0	0	0	0 ± 0	0	0	0	0 ± 0

3C	12496	15407	11677	13193 ± 1132	636	2868	1161	1555 ± 674	0	2469	49	839 ± 814

[1] Gel positions of marked spots is shown in Figure 4.

[2] Volume of each biological replicate.

[3] Mean (± SE) volumes from replicates of *PR*

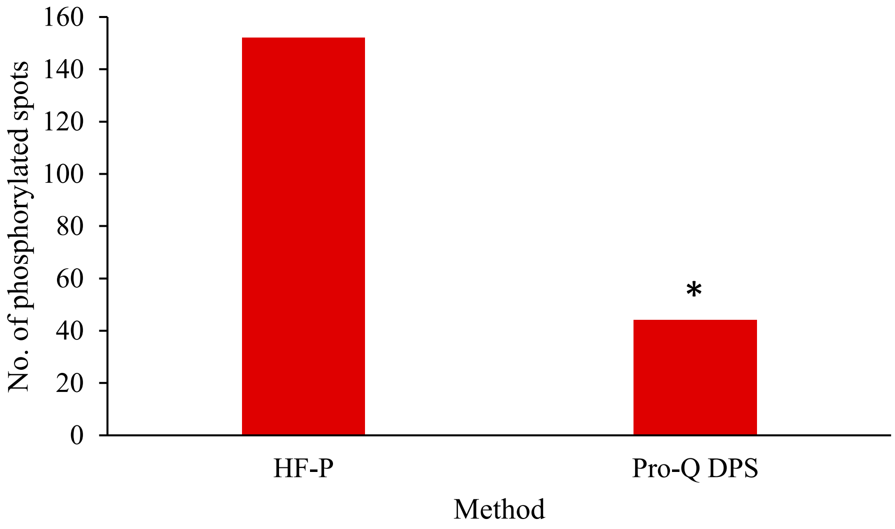

Figure S1. Number of phosphorylated protein spots obtained with HF-P and Pro-Q DPS method (*$P < 0.0001$, Fisher's exact test).
